# Technological Advances to Address Current Issues in Entomology: 2020 Student Debates

**DOI:** 10.1093/jisesa/ieab025

**Published:** 2021-04-28

**Authors:** Lina Bernaola, Molly Darlington, Kadie Britt, Patricia Prade, Morgan Roth, Adrian Pekarcik, Michelle Boone, Dylan Ricke, Anh Tran, Joanie King, Kelly Carruthers, Morgan Thompson, John J Ternest, Sarah E Anderson, Scott W Gula, Kayleigh C Hauri, Jacob R Pecenka, Sajjan Grover, Heena Puri, Surabhi Gupta Vakil

**Affiliations:** 1 Department of Entomology, Louisiana State University, Baton Rouge, LA 70803, USA; 2 Department of Entomology, University of Nebraska-Lincoln, Lincoln, NE 68583, USA; 3 Department of Entomology, Virginia Polytechnic Institute and State University, Blacksburg, VA 24061, USA; 4 Department of Entomology and Nematology, University of Florida, Fort Pierce, FL 34945, USA; 5 Department of Entomology, The Ohio State University, Wooster, OH 44691, USA; 6 Department of Entomology, University of Minnesota, St. Paul, MN 55108, USA; 7 Department of Entomology, Texas A&M University, College Station, TX 77843, USA; 8 Department of Entomology and Nematology, University of Florida, Gainesville, FL 32608, USA; 9 Department of Forestry and Natural Resources, Purdue University, West Lafayette, IN 47907, USA; 10 Department of Entomology, Michigan State University, East Lansing, MI 48824, USA; 11 Department of Entomology, Purdue University, West Lafayette, IN 47907, USA

**Keywords:** locust swarms, genetically modified crops, DNA barcodes, biological control, morphology

## Abstract

The 2020 Student Debates of the Entomological Society of America (ESA) were live-streamed during the Virtual Annual Meeting to debate current, prominent entomological issues of interest to members. The Student Debates Subcommittee of the National ESA Student Affairs Committee coordinated the student efforts throughout the year and hosted the live event. This year, four unbiased introductory speakers provided background for each debate topic while four multi-university teams were each assigned a debate topic under the theme ‘Technological Advances to Address Current Issues in Entomology’. The two debate topics selected were as follows: 1) What is the best taxonomic approach to identify and classify insects? and 2) What is the best current technology to address the locust swarms worldwide? Unbiased introduction speakers and debate teams began preparing approximately six months before the live event. During the live event, teams shared their critical thinking and practiced communication skills by defending their positions on either taxonomical identification and classification of insects or managing the damaging outbreaks of locusts in crops.

The Student Debates competition is an annual meeting tradition of the Entomological Society of America (ESA). It is an energetic event wherein teams from different universities defend specific topics of current interest to the entomological community. This event is organized and hosted by the Student Debates Subcommittee (SDS) of the Student Affairs Committee (SAC). A student can participate as a team member or provide an unbiased introduction to each main topic. Teams will have opposing stances under the assigned topic, which they will prepare for throughout the year. For more specifics on the flow and rubric of the debates, refer to Parker et al. (2019).

Due to the COVID-19 pandemic that began in spring 2020, the ESA annual meeting was held virtually this year, and as such, the Student Debates were live streamed; this new format resulted in a large and engaged audience. The unique circumstance also inspired the formation of unprecedented teams and introductory speakers, represented by a combination of multiple universities as well as new universities that had not participated in the Student Debates in previous years. For the 2020 virtual annual meeting, the theme was ‘Entomology for All’, which the SDS used as a template to devise the central premise for this year’s debate: ‘Technological Advances to Address Current Issues in Entomology’. The SDS chose this theme to promote discussion of current entomological issues of interest to all ESA members and highlight the competing approaches available to address them.

We live in an exciting time full of novel technologies able to solve a range of issues in entomology. Although insects play a large role in our lives, it can be challenging to determine the best methods for studying species and managing pests. By combining tools of morphological characterization, molecular biology, and genetics, we as scientists can better understand insect issues related to taxonomic identification and classification as well as their damaging outbreaks in crops, such as globally experienced locust swarms (Rosenberg and Burt 1999, Zhang et al. 2019). It is critical to not only conduct sound research but to also communicate it effectively to stakeholders and the greater public, which is a key objective of these debates. In order to raise awareness of current entomological issues, the following debate topics selected were as follows:

1) What is the best taxonomic approach to identify and classify insects?
2) What is the best current technology to address the locust swarms worldwide?

The purpose of this article is to summarize the 2020 student debates by first including the unbiased introduction for each topic followed by responses from opposing teams. This event encourages students to work collaboratively and engage in a unique and challenging way by debating topics relevant to entomology. The SAC hopes that ESA members continue to attend this event in upcoming years, taking the time to challenge debaters with questions to further improve their critical thinking skills and the quality of the debates.

## What Is the Best Taxonomic Approach to Identify and Classify Insects?

### Unbiased Introduction by Morgan Roth and Molly Darlington

Insect taxonomy lies at the heart of entomology, and for hundreds of years, insect classification was rooted in morphological observations (Winsor 1976). Insect morphology is a reliable standard upon which numerous books and dichotomous keys are based and has led to the identification of over one million insect species thus far (Stork 2008). Nevertheless, it is estimated that millions of insect species have yet to be classified (Gaston 1991, Stork 2008). Although entomologists of the past did not have a choice regarding classification methods, the development of molecular techniques over the last few decades now offers an alternative to morphology-based taxonomy. The question then remains, which method is best?

Humans have classified animals for centuries, evidenced by Aristotle’s *Historia Animalium* published in the fourth century BC (Aristotle 350BC). This book contains one of the oldest surviving insect classification systems, utilizing physical characteristics for correlation purposes (von Lieven and Humar 2008). Throughout the following millennia, scholars continued to search for morphological patterns within the natural world, adopting new technologies as the years progressed, including the printing press and optics innovations (Engel and Kristensen 2013). Aldrovandi (1602) wrote the book *De Animalibus Insectis Libri VII,* which was the first text exclusively dedicated to entomology and provided a summary on insect morphology, and Ray (1710) who wrote *Historia Insectorum* was an early attempt to develop a classification system based on morphology (Engel and Kristensen 2013). Any brief history of insect taxonomy would be incomplete without mentioning Carl Linnaeus’s *Systema Naturae*, which reorganized Insecta into seven orders based on morphological characteristics (Linnaeus 1758). Finally, in the 19th century, the cladistic revolution popularized by Hennig (1953) introduced the ideas of shared, derived characteristics and hierarchical evolutionary relationships into taxonomy, which still inform methodologies today (Engel and Kristensen 2013). Throughout the centuries, countless other women and men spent their livelihood comparing morphological characteristics, providing a foundation for insect taxonomy we utilize today.

Morphology-based taxonomy has a rich history and has been referenced as the standard for insect classification for centuries. However, scholars have routinely utilized technological innovations to classify insects over the years, and the molecular revolution of the twentieth century was no different. The term ‘molecular biology’ was first used in 1938 (Weaver 1970) and forever changed our understanding of the natural world, along with our ability to identify and classify evolutionary relationships. Since the first mention of DNA barcoding for insect identification in 2003 (Hebert et al. 2003), DNA barcoding has been applied to numerous insect orders and is being used to compile databases in which biodiversity data and genetic information can be overlaid to help further knowledge of species richness (Wilson 2012). Other methods, such as restriction fragment-length polymorphism (RFLP), amplified fragment length polymorphisms (AFLP), and especially molecular phylogenetics have benefited from advancements in computational speeds, high-throughput sequencing, and bioinformatics (Fitch and Margoliash 1967, Sperling et al. 1994). Utilization of molecular techniques is just the most recent advancement in a long and storied history of incorporating novel ideas and technologies to enhance the identification and classification of the natural world around us.

Those in favor of morphology-based classification assert expertise is needed to utilize molecular methods, while morphology remains easily accessible, with international standards and large databases available (Wägele et al. 2011). Additionally, morphology-based methods facilitate citizen science, encouraging collaboration between scientists and the general public, broadening interest in entomology. Proponents of molecular approaches note these methods are useful when classifying cryptic species, yield quick results, are steadily becoming more affordable, and foster multidisciplinary collaborations (DeSalle and Goldstein 2019). Ultimately, both molecular and morphology-based classification offer useful ways to achieve the same goal, and it remains to be seen which method will prevail in the future of insect taxonomy.

## Team 1 Stance: Morphological Characters are the Best Taxonomic Approach to Identify and Classify Insects

### Team Members: Adrian Pekarcik, Michelle Boone, Dylan Ricke, Anh Tran

#### Faculty Advisor: Dr. Sujaya Rao, University of Minnesota

Taxonomy, the description, classification, and naming of organisms, is the foundation of modern biological classification (Wägele et al. 2011). Morphological identification as the basis for taxonomy has been relied upon by humans of all ages and professions, regardless of location or socioeconomic status, for millennia (Matthews 1988). Globally, taxonomists have developed keys and guides, including descriptions of external morphology characters and images, that have facilitated on-site identification of insects. Identification based on morphological characters is especially important for pest species, many of which are cosmopolitan, as successful management requires quick and proper identification (Ramani 2013, Dara 2019, Gagné et al. 2019). While molecular techniques for insect identification are becoming more prevalent, they cannot replace identification using morphological characters.

The taxonomic approach with morphological identification has been sustained over centuries, as it can evolve and incorporate technological innovations and analytical approaches. Novel imaging technologies and applications (e.g., machine vision, scanning electron microscopy, nuclear magnetic resonance imaging) have aided in accurate insect identification and classification, and the digitization of historic biological collections has increased the accessibility of these once exclusive resources (La Salle et al. 2009, Wipfler et al. 2016, Short et al. 2018, Valan et al. 2019). These innovations have resulted in global online databases that allow users to upload specimen images facilitating community science programs between the public and researchers. For example, iNaturalist currently has over 5 million taxonomic records, while the Early Detection and Distribution Mapping System in North America lets users report occurrences of invasive insects based on morphological characteristics (Acorn 2017). Morphological identification resources for insects are also readily available in developing countries (Helmy et al. 2016). The expansion of new technologies has the potential to identify new taxa from digitized collections, community science projects, and to arrange species phylogenetically (Valan et al. 2019).

Although morphological identification of new species requires taxonomic expertise and can take 6 mo from sample collection to identification, it is more economical and convenient than alternative approaches like DNA sequencing that promise ‘rapid-identification’ of species (Collins and Cruickshank 2013). Molecular identification is not a definitive means of demarcating species since it is based on degrees of sequence similarity and is ultimately rooted in morphological verification of physical specimens based on dichotomous traits which can account for variation (including phenotypic plasticity) within the proposed species (Will and Rubinoff 2004). Thus, genetic information is another line of evidence in species delimitation but not a replacement of morphological traits (De Queiroz 2007). Sequencing is largely inaccessible in many developing countries as it is rapidly evolving, requires specialized training, access to the relevant literature, and genomic and computational facilities (Helmy et al. 2016). Overall, molecular methods are estimated to cost 1.7–3.4 times more than morphological techniques per species (Stein et al. 2014).

The identity of a species based on molecular analyses alone can be tenuous, as the results may be skewed by the inappropriate use of neighbor-joining trees, bootstrap resampling, fixed distance thresholds, interpretation of the barcoding gap (Meyer and Paulay 2005, Collins and Cruickshank 2013), and limitations with DNA barcode repositories like the National Center for Biotechnology Information (NCBI) and the Barcode of Life Data Systems (BOLD) (Kvist 2013). For example, 42% of invasive insects were missing from BOLD in a study assessed by Kvist (2013). Additionally, phylogenetic analyses are influenced by the prevalence of recent speciation events, paraphyly (~23% of animal species), interspecific hybridization, often poorly established taxonomy, and high infection by endosymbiotic bacteria like *Wolbachia* whose DNA can impede the replication, or detection, of the target (i.e., insect specimen) sequence during polymerase chain reaction (Meyer and Paulay 2005, Virgilio et al. 2010). In a review of 184 phylogenetic studies from 1977 to 2008, Schlick-Steiner et al. (2010) found that morphological techniques would have identified species more successfully than molecular techniques when used in isolation. Thus, molecular identification should continue to serve as a complement to, but not a replacement of, morphological identification (Wiens 2004).

## Team 2 Stance: Molecular Techniques Are the Best Taxonomic Approach to Identify and Classify Insects

### Team Members: Joanie King, Kelly Carruthers, Morgan Thompson

**Faculty Advisor: Dr. Juliana Rangel, Texas A&M University**

Molecular techniques are the ideal approach to identify and classify insects, offering unique advantages compared with morphology. Insect identification and classification are crucial for insect taxonomy and help form the basis of all entomological fields. Designating and placing specimens into an evolutionary context informs how entomologists approach scientific studies that span from genetic mechanisms to ecosystem functions. Accurate identification and classification of insects remain challenging, particularly because they are a hyperdiverse group of relatively small organisms (Kjer et al. 2016a).

Molecular techniques defy the limitations of morphological identification for correctly assigning a unique taxonomic name to a species. Molecular techniques are ideal for insect species that are challenging to identify by sight (Batovska et al. 2016), such as damaged, dry-pinned or decayed specimens, insect parts, exuviae, insect frass, insect gut content, or sibling species. For such specimens, mitochondrial DNA analyses and use of other molecular markers, such as detecting variations in the conserved cytochrome c oxidase subunit I (COI) genes, can provide accurate identification (Mandal et al. 2014, Batovska et al. 2016), but it is important to note that COI genes are highly conserved in some species and not others (Doorenweerd et al. 2020). Similarly, cryptic species possess no distinctive morphological traits but can be differentiated with DNA barcoding (DeSalle and Goldstein 2019). DNA barcoding is an important but controversial molecular technique, often critiqued for reduced identification accuracy in certain insect groups due to limited numbers of reference specimens (DeSalle and Goldstein 2019). However, rapid increases in the amount of data generated through DNA barcoding, as well as growing use of multiple gene regions for identifications and phylogenetic studies, are predicted to overcome these restrictions and expand the use of DNA barcoding for insect identification and classification (Rota et al. 2016). DNA barcoding may also inform interim taxonomic references before formal names are available, especially for diverse and/or understudied taxa (Novotny and Miller 2014, Meierotto et al. 2019, Zamani et al. 2021).

DNA barcoding can help identify pests and endangered species, leading to effective management strategies (Singh et al. 2016, Spadaro et al. 2020). Throughout the history of ecosystem management, incorrect identification often led to management oversight, such as failure to identify invasive species (Mandal et al. 2014). Invasive species are often managed with natural enemies from their native range. However, rearing out and identifying certain natural enemies, such as endoparasitoids, can be costly and time consuming. DNA barcoding offers the unique advantage of allowing for the identification of endoparasitoids without having to rear them out of hosts (Novotny and Miller 2014). Moreover, molecular techniques can identify insects at the egg stage (Batovska et al. 2016), a crucial advantage relative to morphology, as most immature insect stages cannot be identified by sight. For management of insect vectors of human pathogens, DNA barcoding can be a useful tool for identifying pests such as mosquitos, which are obtained through geographic surveys (Batovska et al. 2016) and reveal novel taxa as possible important vectors of pathogens like malaria (Mandal et al. 2014).

To classify insects, defined as accurately placing specimens within a phylogeny, molecular techniques offer advantages relative to morphology. For instance, phylogenetic modeling is the key method for determining monophyly (Misof et al. 2014). Additionally, studies using a variety of molecular techniques, including but not limited to mitochondrial COI genes, have resolved otherwise indiscernible phylogenies (Mandal et al. 2014, Doorenweerd et al. 2020). Notably, improvements in molecular technologies have resolved longstanding taxonomic issues that arose from both morphological and molecular data, such as the classic ‘Strepsiptera problem’ (Kjer et al. 2016b). Recent genomic research has unraveled how developmental and phylogenetic processes change over time and across lineages (Kjer et al. 2016a). When there are no useful morphological characteristics for a particular insect group, molecular techniques are useful for family- or species-level classification. Furthermore, molecular techniques can identify instances of convergent evolution, such as eusocial behaviors and caste systems among Hymenoptera (Berens et al. 2015).

A final advantage of using molecular techniques for insect identification and classification is the fact that entomology is a data-rich discipline. For example, data for identification of various prey species using barcoding of predator gut content is something that is now possible that was not feasible with observational and morphological techniques (Symondson 2002). Data collected through molecular techniques can aid sampling efforts for trophic food web studies (Novotny and Miller 2014). Furthermore, the use of DNA data and other molecular techniques such as phylogenomics can help overcome problems related to single-specimen or locality species (Deng et al. 2019), generating more connections among data sets. In conclusion, molecular techniques are superior to morphology for the identification and classification of insects.

## What Is the Best Current Technology to Address the Locust Swarms Worldwide?

### Unbiased Introduction by Kadie Britt and Patricia Prade

Insect pests are a serious problem for global agriculture because of feeding damage and increased production costs due to management (Krall and Herok 1997). Certain insect pests, like locusts, have caused agriculture issues since the start of agrarian civilizations (Enserink 2004, Wang et al. 2014) and are often present in swarming populations. Locust swarms occur across a diverse range of global landscapes, including Africa, the Arabian Peninsula, parts of South West Asia, the Caribbean, and South America (Kennedy 1951, Rosenberg and Burt 1999); swarms can cover vast areas, some of which are inaccessible by humans, uninhabitable, dangerously rocky, or covered with thorns (Enserink 2004). It may make sense to manage locusts with conventional pesticides; however, due to the seasonality of swarming locust outbreaks, costs associated with management, frequency of applications, and risks to human and animal health, the use of pesticides is not a recommended or viable option to manage locust swarms in all affected areas (Enserink 2004). Therefore, a need exists for an integrated management program. The implementation of genetically modified (GM) crops that are tolerant or resistant to locusts and the use of biological control agents and/or mycoinsecticides are two potential options. However, the positive and negative outcomes from both methods must be considered.

The use of GM crops to manage unwanted insect pests first occurred in 1996 when the main targets were coleopteran and lepidopteran species (Romeis et al. 2019). Since this work began, the list of target pests has expanded and studies focused on nontarget effects have occurred to provide broader knowledge of use and selection of GM crops (Romeis et al. 2019). Research studies have shown that GM crops can be toxic to locusts and can provide sufficient management levels (Quesada-Moraga and Santiago-Álvarez 2001, Song et al. 2008). However, the research and development required for new GM crop varieties is costly, requires specially equipped laboratories, and can take several years, making GM crops not readily available when needed (Song et al. 2008). Research into new strains of *Bacillus thuringiensis* (Berliner) (Bacillales: Bacillaceae) (*Bt*) may not always be a priority for research groups, and the development of new strains is usually targeted toward other pests of economic concern (Lepidoptera, Coleoptera), not locusts (Song et al. 2008).

The use of biological control, especially as part of an integrated pest management (IPM) program, is potentially viable. While classical biological control is beneficial, upfront research costs can be expensive and time-consuming when solutions are needed immediately. Mycoinsecticides show promise for managing insects from the Acrididae family. For example, Bateman et al. (1996) studied effects of *Metarhizium acridum* (Driver & Milner) (Hypocreales: Clavicipitaceae) on adult locusts to develop a locust-specific, environmentally benign formulation that is compatible with existing application equipment. Under laboratory conditions, Hunter (2005) found that *M. acridum* causes 90–95% mortality in locusts within 6–10 d of application during warmer days with temperatures ranging from 36 to 40°C. Mild spring temperatures ranging from 20 to 30°C can slow the activity of mycoinsecticides with locust mortality occurring 10–14 d after application. While there are issues with mycoinsecticide efficacy and timing of activity, their use in environmentally sensitive areas early on in an outbreak is less expensive than not managing locust outbreaks at all (Hunter 2005). If left unmanaged, swarming populations may eventually need frequent applications of synthetic insecticides when locust numbers have increased substantially and are directly threatening crops.

While we are only highlighting two methods of locust management, all viable options have positive and negative aspects. However, if management methods are combined in an integrated pest management plan, short- and long-term solutions to manage locust outbreaks may be closer than expected.

## Team 3 Stance: Genetically Modified Crops Is the Best Current Technology to Address the Locust Swarms Worldwide

### Team Members: John J. Ternest, Sarah E. Anderson, Scott W. Gula, Kayleigh C. Hauri, Jacob R. Pecenka

#### Faculty Advisor: Dr. Rachel Mallinger, University of Florida

Locusts are devastating pests able to form massive swarms and can ravage millions of hectares of crops across multiple continents. Their rapid, unpredictable dispersal and polyphagous diet create unique management challenges and potential conflicts when coordinating control efforts (Zhang and Hunter 2017, Gay et al. 2020). This problem is exacerbated by global climate change, which accelerates breeding and nymph development and makes predictive modeling and early outbreak responses increasingly challenging (Salih et al. 2020). Long-term solutions for controlling locust swarms must incorporate ease of implementation for farmers, low cost, accessibility, and effective management, which can all be achieved with the use of genetically modified (GM) crops.

Although highly effective GM crops such as those that utilize *Bt* have not been widely used against orthopterans, strains with high toxicity to adult and juvenile migratory locusts have been identified (Song et al. 2008). The use of GM crops in numerous agricultural systems worldwide has led to reduced insecticide use and increased yield, profits, and environmental benefits. The implementation of *Bt* maize in the United States suppressed pest populations regionally, which conveyed protection to surrounding non-GM crops and reduced the need for pesticide applications in the area (Dively et al. 2018). Pest-specific *Bt* cotton varieties in China effectively reduced aphid density without adverse effects on beneficial insects, which increased in abundance (Lu et al. 2012). This pattern is consistent across crops and countries: a 2014 meta-analysis found, on average, that GM crop adoption reduced chemical pesticide use by 37% and increased profits by 68% (Klümper and Qaim 2014). Notably, yield and financial benefits are greatest for small farmers in developing countries, historically, the demographic most impacted by locust swarms (Carpenter 2010, Klümper and Qaim 2014). Genetically modified crops empower individual farmers to effectively limit damage from local locust outbreaks while also providing increased yields, higher economic performance of crops, and lower adverse health effects than conventional pest management strategies (Carpenter 2010).

Conversely, biological and cultural control methods are expensive, labor-intensive, difficult to apply in a precise and timely fashion, and are prone to fail in the field (Wilson et al. 2002, Chandler et al. 2011, Zhang and Hunter 2017, Gay et al. 2020). This is particularly true for biopesticides, which typically have slow kill rates and are highly susceptible to degradation (Chandler et al. 2011). Additionally, widespread use of biopesticides is currently impossible because fluctuating demand for these products makes large-scale production economically infeasible (Chandler et al. 2011). Cultural control methods may require drastic habitat and agricultural modifications (e.g., replacement of crops and natural host plants) to be effective against locusts; however, the long-term economic, ecological, and social consequences of these radical changes have not been fully assessed (Zhang and Hunter 2017). Management strategies reliant on early, consistent monitoring and rapid response are rendered completely ineffective by the lack of international cooperation (Gay et al. 2020). Accurately predicting locust outbreaks is especially difficult because some breeding regions are inaccessible or too large for effective monitoring (Gay et al. 2020, Salih et al. 2020). When these management options fail, the costs are borne by individual farmers left with few control alternatives.

The prophylactic protection provided by GM crops is superior to unproven cultural and biological control methods for managing unpredictable and highly mobile swarms of locusts. Researchers have been promising results from biocontrol for decades, but the technology has not delivered. GM crops offer a real solution: increased yield and profits for growers, targeted and effective control, and environmental benefits through reduced pesticide use. Therefore, GM crops are the best current technology to manage locust swarms reliably and effectively.

## Team 4 Stance: Biological/Cultural Control Is the Best Current Technology to Address the Locust Swarms Worldwide

### Team Members: Sajjan Grover, Heena Puri, Surabhi Gupta Vakil

#### Faculty Advisor: Dr. John Ruberson, University of Nebraska-Lincoln

Locusts occur on most continents: Asia, Africa, Australia, South America, and previously in North America. The rapid development of locust populations and their polyphagous and voracious feeding habits make them challenging insect pests (Zhang et al. 2019). Although locust populations are limited most years, devastating outbreaks are sporadically triggered by high population growth and crowding under certain weather and food conditions (Zhang et al. 2019). The capacity to monitor nascent locust populations has shifted priority from curative to preventative approaches, utilizing slower-acting tools to delay population growth and swarming (Hunter 2010, Zhang et al. 2019). Because chemical insecticides are accessible, cheap, and fast-acting, they have been the dominant tool for both preventative and curative locust control. However, the adverse effects of chemicals on environmental and human health underscore the need for ecofriendly strategies, such as biological control (Bateman et al. 2017). Biological control utilizes natural enemies of pests to reduce pest populations while minimizing adverse effects on environmental and human health (Lomer et al. 2001). Several organisms, such as fungi, bacteria, nematodes, and parasitoids, are being considered as potential biological control agents.

Species/strains of the naturally occurring entomopathogenic fungus *Metarhizium* have proven efficacious against locusts in laboratory and field conditions: *Metarhizium anisopliae* (Metchnikoff) (Hypocreales: Clavicipitaceae) against Australian plague locust, *Chortoicetes terminifera* (Walker) (Orthoptera: Acrididae); *Metarhizium anisopliae flavoviride* (Gams & Rozsypal) against desert locust, *Schistocerca gregaria* (Forsskål) (Orthoptera: Acrididae); and *Metarhizium anisopliae anisopliae* var*. acridum* (Driver & Milner) against oriental migratory locust, *Locusta migratoria manilensis* (Meyen) (Orthoptera: Acrididae) (Langewald et al. 1997, Hunter et al. 2001, Peng et al. 2008). The microsporidian fungus *Paranosema* (*Nosema*) *locustae* (Canning) (Dissociodihaplophasida: Nosematidae) can cause significant mortality to *L. migratoria manilensis* and avert locust transformation from solitary to gregarious forms (Fu et al. 2010). The effectiveness of *P. locustae* may be enhanced by combining it with *Beauveria bassiana* (Bals.-Criv.) (Hypocreales: Cordycipitaceae), affecting the locust gut microflora (Tan et al. 2020). *Bacillus weihenstephanensis* (Flügge) (Bacillales: Bacillaceae) and *Pseudomonas* sp. (Migula) (Pseudomonadales: Pseudomonadaceae) also have potential as biopesticides against *S. gregaria* but require further evaluation (Mashtoly et al. 2019). Among biopesticides, *Metarhizium* based products have been tested for locust control throughout Africa, Australia, North America, and elsewhere, and their impact on nontarget organisms has been well studied (Bateman et al. 2017). It is unlikely that these biopesticides pose serious short- or long-term hazards to nontarget terrestrial or aquatic organisms (Milner et al. 2002, Maute et al. 2017). Biocontrol agents are slow-acting compared with chemical insecticides and may be heavily influenced by the environment (Bateman et al. 2017). Thus, the use of biocontrol agents is most effective when used preventatively, slowing population growth rather than curatively halting massive outbreaks.

Biological control is environmentally safe and less likely than chemical insecticides to induce resistance in pests (Knols et al. 2010), thereby making it valuable against locusts. Entomopathogens may also generate epizootics, thereby extending suppression of locusts in space and time for broader benefit (Lomer et al. 2001). Entomopathogens may be produced locally, enhancing local economies, and is also imported for the control of invasive pests. The advent of techniques like RNAi and CRISPR-Cas9 makes it easier to rapidly identify more virulent entomopathogens (Fang et al. 2014). Other biological control agents, such as viruses, nematodes, and parasitoids, may also offer great opportunities for biocontrol, but their potential remains to be investigated (Zhang et al. 2019).

Cultural control also may offer locust management options, but there are few examples of effective deployment. Heavy livestock grazing can reduce plant nitrogen content and promote locust populations (Cease et al. 2012). Consequently, practices elevating nitrogen and organic matter may reduce locust populations (Word et al. 2019). Cultural practices can be implemented locally or with coordinated regional effort and can yield permanent results. However, these practices can be costly and their environmental impacts severe (e.g., flooding, forestation, or grazing).

No locust management tool lacks disadvantages and risks, but the use of biological control agents offers significant environmental, human health, and socioeconomic benefits that can outweigh the disadvantages. No single tactic will yield ideal locust control. Instead, programs must integrate multiple tactics into an environmentally and economically sustainable, safe, and socially/culturally acceptable strategy (Hunter 2010). Biological control can be an effective core tactic in the locust management toolbox (Lomer et al. 2001).

### Conclusions

ESA student debates are a proven, effective way of promoting critical thinking, communication, collaboration, and enthusiasm for learning and discussing current entomological topics among student members (Parker et al. 2019, Holt et al. 2020). This year was no exception as the student debates were a successful and well-executed event. Despite the pandemic, this year’s debates encouraged new student participation through the formation of multi-university teams and heightened the awareness of the selected topics. In addition, the virtual format of the 2020 National ESA Meeting led to higher attendance than in previous years, and possibly ever before. This new format will hopefully create more awareness and interest for students to participate in future debates. We hope that the debate topics and stances taken on insect identification methods and locust swarm management expanded viewpoints and encouraged further societal discussions in regard to incorporating novel technologies to address topical issues in entomology. The use of both morphological and molecular techniques that allow accurate taxonomic identification of specimens at the species level is necessary for understanding the diversity of insects, evolutionary relationships, and their role as agricultural pests, providing a valuable resource for effective integrative pest management. Additionally, scientists are calling for the use of innovative transgenic crops or biological control agents to replace broad-spectrum chemical pesticides, known to harm the environment, thus enabling sustainable solutions to combat global locust swarms. SAC continually surveys entomological topics of most significant interest to keep the tradition of the debates alive and enriching for all members. Therefore, considering the virtual format and integrated makeup of this year’s debate teams, the 2020 ESA Annual Meeting theme of ‘Entomology for All’ was absolutely captured. We look forward to future ESA Student Debates.
